# Blood Pressure Monitoring as a Digital Health Tool for Improving Diabetes Clinical Outcomes: Retrospective Real-world Study

**DOI:** 10.2196/32923

**Published:** 2022-02-08

**Authors:** ‪Yifat Fundoiano-Hershcovitz, Dror Bacher, Marilyn D Ritholz, David L Horwitz, Omar Manejwala, Pavel Goldstein

**Affiliations:** 1 DarioHealth Caesarea Israel; 2 Joslin Diabetes Center Harvard Medical School Boston, MA United States; 3 DLH Biomedical Consulting Las Vegas, NV United States; 4 School of Public Health University of Haifa Haifa Israel

**Keywords:** blood glucose, blood pressure, monitoring, digital therapeutic, diabetes, hypertension, app, model, chronic disease, health data

## Abstract

**Background:**

Remote data capture for blood glucose (BG) or blood pressure (BP) monitoring and the use of a supportive digital app are becoming the model in diabetes and hypertension chronic care. One of the goals in chronic condition management is to increase awareness and generate behavioral change in order to improve outcomes in diabetes and related comorbidities, such as hypertension. In addition, there is a lack of understanding of the association between BG and BP levels when using digital health tools.

**Objective:**

By applying a rigorous study framework to digital health data, this study investigated the relationship between BP monitoring and BG and BP levels, as well as a lagged association between BP and BG. We hypothesized that during the first 6 months of BP monitoring, BG and BP levels would decrease. Finally, we suggested a positive association between BP levels and the following month’s BG levels.

**Methods:**

In this retrospective, real-world case-control study, we extracted the data of 269 people with type 2 diabetes (T2D) who tracked their BG levels using the Dario digital platform for a chronic condition. We analyzed the digital data of the users who, in addition to BG, monitored their BP using the same app (BP-monitoring [BPM] group, n=137) 6 months before and after starting their BP monitoring. Propensity score matching established a control group, no blood pressure monitoring (NBPM, n=132), matched on demographic and baseline clinical measures to the BPM group. A piecewise mixed model was used for analyzing the time trajectories of BG, BP, and their lagged association.

**Results:**

Analysis revealed a significant difference in BG time trajectories associated with BP monitoring in BPM and NBPM groups (*t*=–2.12, *P*=.03). The BPM group demonstrated BG reduction improvement in the monthly average BG levels during the first 6 months (*t*=–3.57, *P*<.001), while BG did not change for the NBPM group (*t*=0.39, *P*=.70). Both groups showed similarly stable BG time trajectories (B=0.98, *t*=1.16, *P*=.25) before starting the use of the BP-monitoring system. In addition, the BPM group showed a significant reduction in systolic (*t*=–6.42, *P*<.001) and diastolic (*t*=–4.80, *P*<.001) BP during the first 6 months of BP monitoring. Finally, BG levels were positively associated with systolic (B=0.24, *t*=2.77, *P*=.001) and diastolic (B=0.30, *t*=2.41, *P*=.02) BP.

**Conclusions:**

The results of this study shed light on the association between BG and BP levels and on the role of BP self-monitoring in diabetes management. Our findings also underscore the need and provide a basis for a comprehensive approach to understanding the mechanism of BP regulation associated with BG.

## Introduction

Major goals for the treatment of diabetes are to prevent or delay complications and optimize the quality of life [1]. People with type 2 diabetes (T2D) face challenging self-management regimens to improve glycemia and decrease morbidity and mortality, while often dealing with high costs of care [2]. Hypertension is the most common chronic illness in the United States, and the standard model of office-based care delivery continues to yield suboptimal outcomes, with approximately 50% of affected patients not achieving blood pressure (BP) control [3]. Elevated BP values are a common finding in people with T2D. In fact, hypertension is reported in over two-thirds of patients with T2D [4], and its development coincides with the development of hyperglycemia [5]. Furthermore, a large proportion of persons with diabetes exhibit poorly controlled hypertension, which may reflect not only delayed recognition of the presence of hypertension, clinical inaction, and poor adherence to the prescribed regimen but also uncertainty regarding the treatment targets and pathogenic correlation [6].

The pathogenic relationship between T2D and hypertension is assumed to be bidirectional [7]. Elevated BP levels are supposed to reflect at least partially the impact of the underlying insulin resistance on the vasculature and kidneys, while there is clinical evidence suggesting that disturbances in carbohydrate metabolism are more common in individuals with hypertension [4]. In persons with diabetes, hypertension confers an enhanced risk of cardiovascular disorder, having a similar risk for people with hypertension but without diabetes [5]. Hypertension and diabetes are major risk factors for cardiac diseases, stroke, and kidney disorders [8-10]; however, hypertension is the leading cardiovascular disease–related cause of morbidity and mortality among persons with T2D [11,12].

Previous studies have shown the beneficial effect of BP-lowering treatment on end-stage renal disorders in T2D [13]. Moreover, a significant improvement was demonstrated in all diabetes-related outcomes resulting from long-term tight BP control in patients with T2D and hypertension [14,15].

Among normotensive individuals, T2D at baseline was shown as a significant predictor of incident hypertension (independent of age, body mass index [BMI], and family history of diabetes) [6]. Furthermore, most of the care for the patient with hypertension typically relies on the primary care physician, whose time for face-to-face patient care has become progressively limited [3]. Approximately half of the persons treated for T2D do not have adequate BP or glycemic control [16]. It is clear that management of a chronic condition requires a change in strategy to meet the real-time needs of the population.

Ideal management of chronic conditions, such as T2D and hypertension, often includes monitoring lifestyle changes and pharmacological interventions to improve metabolic health [17]. Home BP measurement has been recommended by many hypertension guidelines and addresses several limitations of traditional office-based care, including reducing misclassification because of white-coat or masked hypertension and an ability to take a more suitable action and a course of corrective therapy [18].

Self-monitoring has been shown in previous studies as 1 of the key elements for successful chronic condition management. One of the examples of successful chronic condition management was shown with self-monitoring behaviors involving weight measurements, which demonstrated self-monitoring as a significant predictor of weight loss during 6 months [19]. Of note, there is evidence showing that patients’ willingness to self-monitor is associated with disease controllability, and persons with diabetes, asthma, and hypertension are most willing to self-monitor [20]. Home glucose self-monitoring has been associated with improved glycemic control and reduced long-term complications [21]. Current meta-analysis supports the claim that self-monitoring can significantly reduce BP [22,23].

Treatment optimization through digital health could enhance users’ alertness to their health conditions through real-time monitoring, leading to effective treatments. Timely communication and feedback also can play a key role in efforts toward achieving hypertension and diabetes control. Technology-driven solutions can help persons with diabetes build awareness of their daily health-related behaviors and promote increased engagement with those behaviors [24-26]. Communication of test results also has been shown to be highly desired among people with hypertension [27], and lifestyle-focused educational messages providing advice, motivational reminders, and support also were shown to be effective in improving hypertension and other chronic conditions [28]. Real-time digital communication can additionally include progress reports focusing on achieving BP and blood glucose (BG) goals and displaying insights as well as guidelines promoting lifestyle change. Using a mobile app for self-management purposes could make it easier for people with chronic conditions to obtain insight into and control of their BG and BP levels.

On the one hand, mobile apps have been shown to improve diabetes outcomes via education and support for adhering to evidence-based recommendations [29-32]. On the other hand, there are mobile apps focused solely on hypertension management. These are designed primarily for health management functions and have proved an effective solution in improving medication adherence and systolic and diastolic BP levels [33-35]. Although mobile apps have the potential to be beneficial for people with hypertension or diabetes, little is known about their efficacy targeting several chronic conditions at 1 time or monitoring both BP and BG levels on 1 mobile app [36]. Further research is necessary for investigating the effectiveness of mobile health for hypertension self-management over time [35]. Moreover, the current literature is missing rigorous real-life studies to test the role of BP self-monitoring and diabetes management and to better understand the direct association between BP monitoring and glycemic outcomes. Further, in glucose-lowering trials, it is not clear whether different ongoing BP levels are associated with different BG-lowering outcome effects [37,38].

This study used a retrospective analysis of a home-use diabetes BG meter and BP-monitoring system with full data capture using a supportive mobile app among people with T2D and poorly controlled BP levels. This study can illuminate the dynamic of the relative contribution of BP self-monitoring to successful diabetes and hypertension self-management using real-life data. We analyzed users’ data for 6 months before and 6 months after using the BP-monitoring system and compared them with a matched control group that never used the BP-monitoring system (NBPM). We hypothesized that BP monitoring will be associated with reduction in systolic and diastolic BP, as well as with BG levels. We additionally hypothesized a linkage between BP levels and following BG levels.

## Methods

### Platform

This study used the Dario digital therapeutics solution (DarioHealth) for chronic conditions to support self-management of diabetes and high BP levels. The Dario BP-monitoring system combines an innovative meter with a phone app that is available for both Android and iOS devices. The glucose meter consists of a small, pocket-size holder for strips, a lancet, and the meter. The BG meter is removed from the holder and plugged directly into a smart mobile device, effectively converting the smart mobile device into a display screen for the meter. The BP-monitoring system measures the systolic and diastolic BP and pulse rate by using a noninvasive technique in which an inflatable cuff is wrapped on the upper arm. The BP-monitoring system provides Bluetooth transmission. The BP cuff is paired with the mobile app, and the data are transmitted to the smart mobile device via Bluetooth. The BP reading is displayed on the mobile app screen.

First, connecting the BG meter directly and pairing the BP cuff to the phone ensures 100% data capture during glucose readings. Second, users open the mobile app with their BG or BP measurement. The measurements are taken independently. This makes contextually tagging a measurement easy at the time of taking the measurement. More specifically, the measurement is shown on the mobile phone in a decision support system view. After the measurement is shown, the user is transferred to a data entry screen where additional information (measurement time [fasting/premeal/postmeal/bedtime]; carbohydrate intake (g); meal, mood, and location settings; and physical activity [kcal]) can be added to the BG measurement. All information is stored in the users’ logbook in the app, attached to the specific BG or BP reading. Data are uploaded to the cloud for backup and further analysis. Mobile app functions include interface design elements as well as specific educational content, wording, or digital interventions that affect the users’ choices in the digital environment that provides personal health information and prompt feedback.

### Measures

The outcome metrics were the monthly average BP level (systolic and diastolic BP), defined as the mean of all the user’s BP measurements taken over a 30-day interval, and the monthly average BG level, defined as the mean of all the user’s BG measurements taken over a 30-day interval.

The relationships of interest potentially could be investigated on different scales emphasizing daily, weekly, or monthly fluctuations. In this real-world data analysis, the timescale was designed to reflect the monthly aggregated interval change over a 6-month period because of the difficulty in tracking daily changes in digital monitoring.

The mobile app collected the following medical and sociodemographic information (by self-report) for each user: gender, age, BMI, physical activity, stress level, and comorbidities, such as high lipids, chronic kidney disease, cardiovascular disease, sleep disorder, cancer, or stress and depression. Socioeconomic status was matched by applying zip code data to census.gov. All data were transferred and stored in compliance with Health Insurance Portability and Accountability Act (HIPAA) requirements, using Amazon AWS database services. All data were anonymized before extraction for this study.

### Users

In total, 269 users with T2D who used the Dario BP-monitoring system between 2019 and 2020 were included in this analysis. The sample included 172 (63.9%) men, 45 (16.7%) having comorbidities. Their average age was 62.0 (SD 11.9) years, average BMI was 31.7 (SD 6.4), and median household income was US $29,100 (SD 3150).

### Study Design

The BPM group included persons with diabetes who measured their BG and BP levels (BPM group, n=137, 50.9%). Inclusion criteria were as follows for the BPM group: measured BP levels in the first and fourth months, with at least 5 BP measurements per month; the first-month average BP level was in the elevated category (systolic BP 120-129 mmHg; diastolic BP less than 80 mmHg) [18] or above; and measured BG levels in the first and fourth months after starting to use the Dario BP-monitoring system, with at least 5 BG measurements per month.

We applied a quasi-experimental case-control study design to improve the methodological rigor and validity of the findings and take advantage of the users’ digital follow-up. We used the existing Dario database to extract the background population to match a control group, NBPM, that did not use BP monitoring. The BG measurement inclusion criterion for the NBPM group was the same as for the BPM group (measured BG levels in the first and fourth months, with at least 5 BG measurements per month) to find the best match for the cohort of 137 (49.1%) users who started using BP monitoring. Matching is used in the context of estimating the causal effect of a binary condition of interested in or exposed to on an outcome, while controlling for potential confounding variables or variables prognostic of the outcome [39]. The goal of matching was to produce a covariate balance, seeking for approximately equal distributions of covariates in the 2 groups, as they would be in a randomized experiment. The covariate balance results in increased robustness to the choice of model used to estimate the treatment effect. The match was based on sociodemographic and clinical parameters: gender, age, BMI, physical activity level, stress level, and self-reported comorbidities (hypertension, high lipids, chronic kidney disease, cardiovascular disease, sleep disorder, cancer, mental condition), socioeconomic status, number of BG measurements, and average BG. We applied the nearest-neighbor propensity score matching without replacement, with the propensity score estimated using logistic regression of the treatment on the covariates [40,41]. This approach resulted in an adequate balance using the data of 132 users who did not measure BP.

No significant differences were found between BPM and NBPM conditions by age (B=–0.007, Z=–0.22, *P*=.83), gender (B=0.53, Z=0.59, *P*=.56), BMI (B=0.03, Z=0.34, *P*=.73), physical activity level (0=not active, 10=very active; B=–0.28, Z=–1.55, *P*=.12), stress level (0=not stressed, 10 =very stressed; B=0.002, Z=0.01, *P*=.99), median household income (B=–0.28, Z=–1.55, *P*=.12), number of BG measurements (B=–0.01, Z=–0.60, *P*=.55), insulin treatment (B=–0.98, Z=–0.90, *P*=.37), comorbidities (B=–0.66, Z=–0.65, *P*=.52), and digital engagement (tagging meal type, physical activity in the context of measurement [26]; B=0.02, Z=0.97, *P*=.33).

Ethical & Independent Review Services [33], a professional review board, issued the institutional review board exemption for this study (#18032-04).

### Analytic Approach

A classical linear longitudinal model assumes a single-slope growth pattern for changes in an outcome variable across time. Sometimes, such a simple model does not fit the empirical data. In contrast, piecewise-based mixed-effects models allow flexibility in the modeling of trajectories across time [42]. Here, a mixed piecewise model assessed differences in the monthly average BG level in 2 segments: before and after BP-monitoring system usage. The piecewise model allowed the data to exhibit different linear trends over their different regions. This statistical approach provided an opportunity to model curvilinear changes in the monthly average BG level as a single process and to test complex effects.

Users’ data were centered around the beginning of the BP measurements and 6 months before and after that point were included in the analysis. For the NBPM (control) group, that had never started BP measurements, we included users with at least 18 months of monitoring, choosing a random cutoff point and including in the analysis only the data collected during 6 months before and after the simulated cutoff point.

A piecewise-based mixed‐effects model was fit to the data, modeling temporal changes of the monthly average BG level for the 2 groups (BPM vs NBPM). The piecewise cutoff point for the model was set to the beginning of BG monitoring, assuming a change in the time-related monthly average BG trajectory between the groups by the included interaction terms between time trajectories and group. The model included a person-based random intercept and random slope for the time trajectory after the piecewise cutoff.

Next, we used mixed-model analysis to access the time trajectory of systolic and diastolic BP for the initial 6 months of BP monitoring. The models included a random intercept and random slope of the time trajectory. We reported unstandardized regression weights (B), test statistics (*t*), and associated significance (*P*).

To better understand the dynamic of BP and BG association, we conducted a lagged analysis, predicting the following month’s BG level based on the BP level.

## Results

### BP Monitoring Is Associated With BG Levels

**Figure 1:**
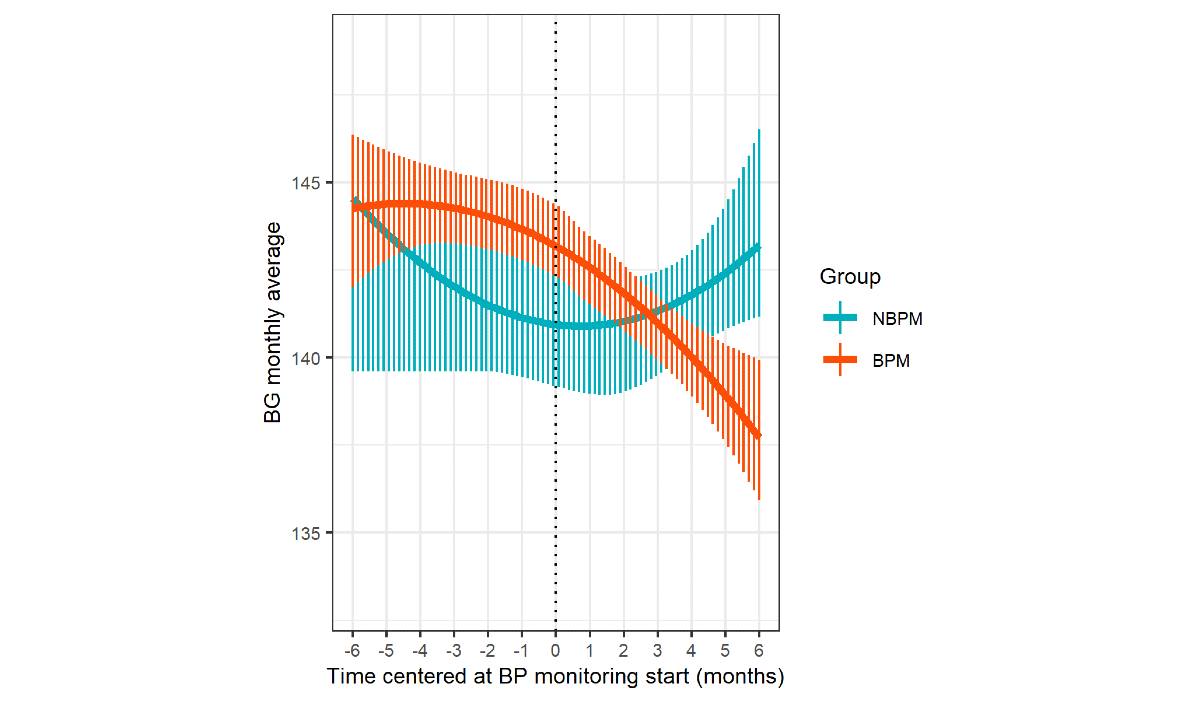
BG monthly average fluctuation for the BPM group and the NBPM group. Zero in the x-axis means the start of BP monitoring. Vertical lines represent a 95% CI over time. BG: blood glucose; BPM: blood pressure monitoring; NBPM: no blood pressure monitoring.

Piecewise mixed-model analysis revealed a significant interaction between the time after starting BP monitoring and group (B=−1.50, *t*=–2.12, *P*=.03) on BG levels. The BPM group showed a significant reduction in BG (B=−1.16, *t*=–3.57, *P*<.001), while the NBPM group did not show a significant time trend (B=0.24, *t*=0.39, *P*=.70); see Figure 1. Before BP monitoring, group difference was observed in BG time trends (B=0.98, *t*=1.16, *P=*.25), both BPM (B=−0.13, *t*=–0.43, *P=*.67) and NBPM (B=−1.16, *t*=–1.49, *P=*.14) groups showed no BG trend. Extended information is provided in Multimedia Appendix 1.

We reran the analysis including all the potential confounders into the models. In the new analysis, the pattern of the findings remained the same. Users with hypertension (B=25.27, *t*=2.57, *P*=.02) and insulin treatment (B=18.17, *t*=3.02, *P*=.003) showed increased monthly average BG levels. In addition, stress level (B=4.26, *t*=2.38, *P*=.02) and median household income (B=0.006, *t*=4.88, *P*<.001) were associated with increased monthly average BG levels. Age, gender, BMI, physical activity, alcohol consumption, and comorbidities were not related to the monthly average BG (all *P*>.09).

### BP Monitoring and the Link to BG

**Figure 2:**
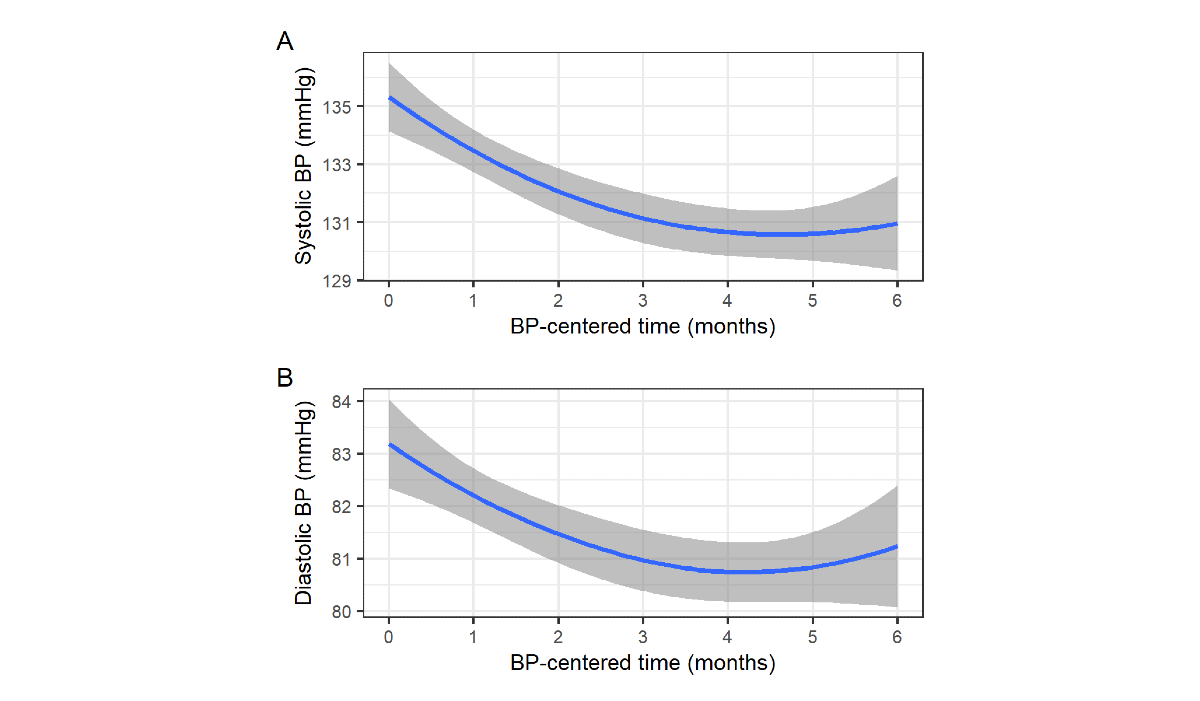
Monthly average BP fluctuation over the first 6 months of monitoring for (A) systolic and (B) diastolic BP. The x-axis presents the centered time, with 0 indicating the start of BP monitoring. The gray area represents a 95% CI over time. BP: blood pressure.

We analyzed the recorded monthly averaged BP during the first 6 months of monitoring. Systolic (B=−0.82, *t*=–6.42, *P*<.001, 135.4-130.8 mmHg) and diastolic (B=−0.41, *t*=–4.80, *P*<.001, 83.3-81.7 mmHg) BP showed significant reductions during that period (Figure 2), and 37 (27%) of 137 users achieved systolic BP reduction of >10 mmHg (*P*<.001). In addition, results from the lagged analysis, predicting the following month’s BG based on BP levels, showed that following month’s elevated BG is associated with higher systolic (B=0.24, *t*=2.77, *P*=.001) and diastolic (B=0.30, *t*=2.41, *P*=.02) BP.

## Discussion

### Principal Results

This study showed that using a piecewise mixed-model statistical framework appears to be an appropriate base model to describe nonlinear fluctuations in BG levels comparing different user cohorts over time. In our study, the model indicated that before the BP adoption phase, both groups evidenced flat trajectories. However, after starting the use of the BP monitoring system connected to the same mobile app, the BPM group experienced a significant decrease in BG levels, while the NBPM group’s BG levels remained flat. In addition, we ran a lagged analysis demonstrating that monthly systolic and diastolic BP can predict the following month’s average BG. This finding suggests the hypothesis that BP reduction may serve as a mechanism of BG reduction; further studies would be needed to confirm this and to analyze the mechanism by which BP reduction has an effect on BG levels.

This real-world analysis presents data analyzing associations between BP monitoring and reduction in BG levels in people with T2D and elevated BP for those who start using the mobile app for diabetes and hypertension management. More specifically, results indicate an association between BG reduction and BP monitoring. This effect was not observed among the users who did not use BP monitoring, although both groups showed statistically equivalent BG trends before BP monitoring and had similar demographic and clinical characteristics. In addition, analysis of the monthly average BP during the first 6 months of monitoring showed a significant decrease in systolic and diastolic BP during this period. Moreover, monthly averaged BG was associated with systolic and diastolic BP levels.

Consistent with the literature, we found that the BPM group, which monitored their BP and improved their levels over time, experienced a change in their BG levels, while the NBPM group’s levels remained flat over the same time. Moreover, based on the lagged analysis, we observed that higher systolic and diastolic BP is associated with elevated BG in the following month. Previous studies have shown a significant improvement in all diabetes-related outcomes resulting from long-term tight BP control in patients with T2D and hypertension [14,15]. There is substantial overlap between T2D and hypertension in etiology and illness mechanisms. Hypertension and diabetes substantially share common pathways, such as obesity, inflammation, oxidative stress, insulin resistance, and mental stress [43,44]. Patients with diabetes experience increased peripheral artery resistance caused by vascular remodeling and increased body fluid volume associated with insulin resistance–induced hyperinsulinemia and hyperglycemia. Both these mechanisms elevate systemic BP [7].

The constellation of metabolically related abnormalities, such as obesity, glucose intolerance, and hypertension, are evident in metabolic syndrome [45]. Successful management should address all the factors involved. The goal in a clinical setting is to improve the ability to identify and intervene with factors contributing to metabolic syndrome, including lifestyle modification, weight management, diet, and physical activity changes. One critical view on the individualization of target goals for patients at risk is that this has not always been well adapted for people with low education levels or other sociocultural factors that could distract from finding the time and motivation for improving their individual lifestyles [46]. Our participants showed elevated BP, BG, and BMI. Such a clinical profile resonates with previous diabetes studies [47]. Due to the nature of metabolic disease, factors such as high BP, diabetes, and obesity are shown to be the most suitable therapy areas to address via digital health. This approach may provide information suitable for the user’s clinical condition and may improve self-management efficiency and the clinical course through personalized intervention [48]. Lifestyle changes, such as dietary habits and following exercise recommendations, are crucial for persons with diabetes and hypertension, even when pharmacological treatment is required [43,49]. Further, guidance on lifestyle changes must be provided for people with diabetes and hypertension.

BP management presents a possible course to control BG and improve the patient’s well-being. Reducing BP is more helpful for people with than without diabetes in terms of absolute cardiovascular risk [43]. Previous studies have revealed the beneficial effect of BP treatment in people with high-risk diabetes: attaining standard glycemic control resulted in a decreased risk of cardiovascular events, including heart failure [50].

Cardiovascular disease is the most significant health threat to adults with T2D, and it is clear that efforts aimed at controlling BP and cholesterol will have much greater effects on health outcomes than those focused only on glycemic control [51]. For reducing cardiovascular risk, treatment should focus on specific goals: reductions in systolic and diastolic BP, BG, and plasma low-density lipoprotein (LDL) cholesterol [45].

Our results also revealed a synergistic effect in that the management of BG and the management of BP became evident with the start of controlling BP levels, while this association was not present in the NBPM group, which did not use the BP-monitoring system. Moreover, we demonstrated that the diabetes clinical outcome (ie, monthly averaged BG) is positively lagged with the hypertension clinical outcome (ie, systolic and diastolic BP levels).

It was previously shown how distinct features of a digital therapeutic app have the potential to deliver equitable person-centric care and how digital engagement can play a key role in enhancing a person’s chronic condition self-management [16,52-54]. Previously, we demonstrated that digital engagement may improve diabetes management [26]. Importantly, in this study, the BPM and NBPM groups were not different in their digital engagement. In addition, the tracking tool can be disseminated via a simple-to-use, accessible, and low-cost device. Further, the median household distribution of the users in both groups was equal and revealed that the digital diabetes management solution is desired and affordable among lower-, middle-, and high-income levels to improve glycemic outcomes.

From a psychological perspective, it is assumed that individuals using a digital platform may develop more active roles in managing their health [55]. Previous studies have identified psychosocial factors that could be targeted by interventions to improve diabetes self-management and treatment outcomes [51]. National survey data from the United States highlight basic knowledge gaps among many adults with diabetes and note that less than 50% know their level of glycemic control and only 63% know their BP level [56]. For people with hypertension, evidence for digital interventions has mostly come from small trials with relatively short follow-up and substantial heterogeneity of results [57]. Thus, more studies are needed with larger samples and longer periods of follow-up. Digital interventions (eg, apps, programs, or health software) have the potential to support people in self-management and to facilitate lifestyle change [57].

Our finding on the significant decrease in systolic and diastolic BP occurring within the first 6 months of using the device and mobile app suggests that even the short-term use of our digital monitoring device may be an effective means to increase users’ knowledge base and self-care behavioral awareness in the context of everyday life. The mobile app provided the users with numeracy skills, including the ability to interpret and respond to hypertension numerical feedback and to focus greater attention on the self-management of more than 1 chronic condition. The intervention is designed to influence user behavior by using a person-based approach. Users were advised with personalized reminders to take BP readings, with specific messages driven by their BP levels, calculated averages, displayed BP measurements levels by color scale, and other lifestyle activities (smoking, caffeine, activity). Health behavior change theory posits that new health behaviors emerge when people gain both knowledge and self-efficacy to implement said knowledge [58-60].

The logbook screen inside the mobile app presenting measurements and data records possibly can be shared with the health care provider for further support. The digitalization of health care has the potential to save time and money and enable better physician-patient relationships and personalized treatments based on the specific characteristics of patients, especially patients with hypertension [61,62]. Important components may play a role in regulation of BP and BG and other self-measured values by health care providers.

Finally, our findings indicated that monitoring several chronic conditions may have the potential to offer a greater means for helping person with diabetes and hypertension effectively modulate their glycemia and BP than managing each of the conditions separately. We expect that the analytical approach applied in this study will be useful for examining other chronic conditions and metabolic syndrome outcomes (eg, lipid profile and weight loss). Moreover, this type of analysis may provide valuable information for optimizing patients’ planning and strategies for chronic condition management.

### Limitations

We noted several limitations in this study. First, as in all studies involving retrospective real-world data, groups were not randomly assigned, and treatment protocols were not prescribed. Both limitations created challenges for drawing casual effects. It is possible that users who chose to manage both diabetes and hypertension were those who were motivated to change. However, our inclusion criteria were designed to ensure that both BPM and NBPM groups showed evidence of being engaged with their diabetes management. There were no significant differences between the groups in terms of the number of BG measurements and digital engagement (behavioral tagging). This would suggest that motivation may not be the primary difference between BPM and NBPM. That said, the statistical modeling covers the pitfalls of the comparison between the 2 groups, allowing a quasi-causal inference. However, there might be variables that were not collected that may impact the group imbalance.

In this real-world data analysis, the timescale was designed to reflect the monthly interval change over the 6-month period before and the 6-month period after starting the use of the BP-monitoring system. However, the relationship of interest in this study could be potentially investigated on different scales emphasizing daily, weekly, or monthly fluctuations. Owing to the difficulty in tracking daily changes in real-world studies, most studies focus on monthly fluctuations.

Another challenge regarding our data was that available demographic data were limited. Although there were no differences between groups by age, gender, or median household income, there is always the possibility that uncontrolled demographic bias was present from other demographic factors. In addition, available medical and physical data were limited. No differences existed between groups in terms of physical activity, stress level, insulin treatment, and other comorbidities. However, there is always the possibility that an uncontrolled parameter bias was present from other medical record factors.

### Conclusion

Our findings show a significant association between BP measurement and improved glycemic control. The association is consistent with the hypothesis that there is a physiologic link between BP and glucose control. An alternative explanation is that persons who measure both parameters are more likely to be involved and motivated in their health care. Focusing on BP self-monitoring and lifestyle activities may lead to better glycemic outcomes. The clinical impact was observed in users who measured their BG in the first 6-month period of BP monitoring. We also observed real-time linkage between a reduction in BP levels and a reduction in BG levels. From the behavioral science perspective, this is not surprising. Simultaneously, directing effort onto actionable areas for improvement of BP is likely to increase the thought and action needed for improvement of BG. Moreover, the process of BP self-monitoring in lowering systolic and diastolic BP levels was demonstrated. Future work should focus on investigating the mechanisms underlying the comorbidity of diabetes and hypertension and their management, identifying and applying mediation models and behavioral interventions that go beyond actionable multiple chronic conditions that drive prohealth behavioral change. Furthermore, similar studies examining the impact of gradual trajectories on other behavior changes, including health coaching, gamification, and behavioral economics, are essential. These investigations would help move the field beyond the claim of “what is the impact of the digital tools on managing chronic conditions such as diabetes or hypertension” to a deeper understanding of how digital solutions drive clinical outcomes and how to integrate multiple digital solutions and under what clinical situations. Finally, qualitative research is needed for understanding users’ real-world experiences and as a tool for sensitivity analysis and validation of complex computational models in order to enhance personalized medical approaches.

## Appendix

Multimedia Appendix 1 Piecewise mixed-model analysis of BP monitoring affecting monthly BG. BG: blood glucose; BP: blood pressure.

## Abbreviations

BG: blood glucose
BP: blood pressure
BMI: body mass index
BPM: blood pressure monitoring
HIPAA: Health Insurance Portability and Accountability Act
LDL: low-density lipoprotein
NBPM: no blood pressure monitoring
T2D: type 2 diabetes
