# Supplementary material for: Blood Pressure Monitoring as a Digital Health Tool for Improving Diabetes Clinical Outcomes: Retrospective Real-world Study
Source: J Med Internet Res. 2022 Feb 8;24(2):e32923. doi: 10.2196/32923 (PMC8864523; doi:10.2196/32923)
Supplement: Multimedia Appendix 1 [file jmir_v24i2e32923_app1.docx]

Multimedia Appendix 1: **Piecewise mixed model analysis of the BP monitoring affecting monthly BG.**

|  |  | | | |
| --- | --- | --- | --- | --- |
| *Predictors* | *Estimates* | *CI* | *T Statistic* | *P* |
| (Intercept) | 139.00 | 132.50 – 145.51 | 41.87 | <0.001 |
| time1 | -1.01 | -2.54 – 0.51 | -1.30 | 0.194 |
| time2 | 0.22 | -0.77 – 1.22 | 0.44 | 0.660 |
| group [BPM] | 2.46 | -6.23 – 11.16 | 0.56 | 0.579 |
| time1 * group [BPM] | 0.98 | -0.67 – 2.64 | 1.16 | 0.245 |
| time2 * group [BPM] | -1.50 | -2.89 – -0.11 | -2.12 | **0.034** |
| *Random Effects* | | | | |
| σ^2^ | 309.40 | | | |
| τ_00_ _uid_ | 1108.30 | | | |
| τ_11_ _uid.time2_ | 19.40 | | | |
| ρ_01_ _uid_ | -0.47 | | | |
| ICC | 0.76 | | | |
|  |  | | | |

* σ^2^ – represents model residuals, τ_00_ , τ_11_ and τ_12_ are random intercept, and random slopes for the time trajectories during 1-6 months and 7-12 months correspondingly.
